# Photochemical Redox Cycling of Naphthoquinones Mediated by Methylene Blue and Pheophorbide A

**DOI:** 10.3390/molecules30061351

**Published:** 2025-03-18

**Authors:** Lisa M. Landino, Joseph A. Reed

**Affiliations:** Department of Chemistry, College of William & Mary, Williamsburg, VA 23185, USA

**Keywords:** naphthoquinone, benzoquinone, vitamin K, singlet oxygen, photoreduction, chlorophyll, pheophorbide A, methylene blue, hydrogen peroxide

## Abstract

The photoreduction of plastoquinone, a para-benzoquinone, by chlorophyll initiates photosynthesis in chloroplasts. The direct photoreduction of biologically relevant quinones by dietary chlorophyll metabolites has been reported and may influence health outcomes. We examined red light-mediated photoreduction of ortho- and para-naphthoquinones including vitamin K_3_ using the photosensitizers methylene blue and pheophorbide A, a chlorophyll metabolite. Naphthoquinone reduction was monitored by UV/Visible spectroscopy and required a photosensitizer, red light and a tertiary amine electron donor. Combinations of methylene blue and ethylenediaminetetraacetic acid or pheophorbide A and triethanolamine in 20% dimethylformamide were employed for all photoreduction experiments. Hydrogen peroxide was generated during the photochemical reactions by singlet oxygen-dependent oxidation of the reduced naphthoquinones. Hydrogen peroxide was quantified with horseradish peroxidase following irradiation; the reduced naphthoquinones acted as peroxidase co-substrates. Histidine, a singlet oxygen scavenger, enhanced the rate of photoreduction by limiting the re-oxidation process. Catalase slowed the rate of photoreduction by regenerating molecular oxygen from hydrogen peroxide so that it could be photoexcited to singlet oxygen. The rates and extent of naphthoquinone photoreduction were dependent on molecular oxygen exposure in different reaction formats including in a cuvette and a plate well. Reduction of the tetrazolium salt MTT to the formazan via electron transfer from the photoreduced quinones was also used to quantitate the extent of photoreduction.

## 1. Introduction

Chlorophyll, the light-harvesting pigment of photosynthesis, is the most abundant photosensitizer on Earth [1]. Photoexcitation of it by red light initiates electron transfer to plastoquinone, a para-benzoquinone that is embedded in chloroplast membranes of plant cells [2]. The ultimate end-products of electron transfer in chloroplasts are glucose and molecular oxygen (O_2_).

While photosynthetic steps have been examined in great detail, the photochemical properties of free chlorophyll and its dietary metabolites have received much less scrutiny. Yet recent research shows that dietary chlorophyll metabolites accumulated in multiple cellular locations including the brain, gut and fat of mice [3]. These chlorophyll metabolites in mice were still photo-excitable and detectable because of their characteristic fluorescence emission.

Ubiquinone (also known as CoQ_10_), like plastoquinone, is a membrane-soluble para-quinone that cycles between reduced and oxidized forms in the mitochondrial electron transport chain (ETC) [4,5]. Qu et al. showed that the combination of chlorophyll metabolites and red light reduced ubiquinone to ubiquinol in vitro [6]. Because the ETC generates ATP, chlorophyll- and red light-initiated photoreduction of ubiquinone suggested a novel mechanism that increases ATP synthesis. In support of this, when pyropheophorbide A (ppA), a chlorophyll metabolite, was combined with intact mouse mitochondria and the mixture was exposed to red light, ATP yield increased [3]. When similar experiments were performed in *C. elegans*, both ATP synthesis and life span increased [7].

Although it is clear from these studies that ubiquinone is a likely target for photoreduction by chlorophyll metabolites and red light, we hypothesized that other cellular components are possible photoreduction substrates. Catechols comprise a large set of molecules including the plant antioxidants catechin and EGCG from green tea and the neurotransmitters epinephrine and dopamine. Catechol antioxidants scavenge reactive oxygen species (ROS) that accumulate as normal byproducts of electron transfer to O_2_ in the ETC [8,9,10].

When catechols scavenge ROS, they are oxidized to o-quinones, transient species that resemble plastoquinone and ubiquinone. In Parkinson’s disease, oxidation of dopamine to the corresponding o-quinone results in protein cysteine modification in the substantia nigra [11,12]. The photoreduction of an o-quinone back to its catechol form by the combination of red light and chlorophyll metabolites would be a novel mechanism for increasing antioxidant capacity and possibly limit protein damage in Parkinson’s disease. A light requirement for optimal health is well established; ultraviolet light is required for vitamin D biosynthesis [13].

We reported that dopamine can be oxidized to its o-quinone intermediate by tyrosinase or by singlet oxygen (^1^O_2_)-induced photo-oxidation and then photoreduced by the combination of the same photosensitizer, electron donor and red light [14]. However, these studies were challenging because the photo-oxidation of dopamine to o-quinone by ^1^O_2_ and photoreduction back to catechol occurred concurrently. These reactions were observed with both methylene blue (MB), a well-characterized chemical photosensitizer, or with pheophorbide A (pheoA), a chlorophyll metabolite.

**Scheme 1:**
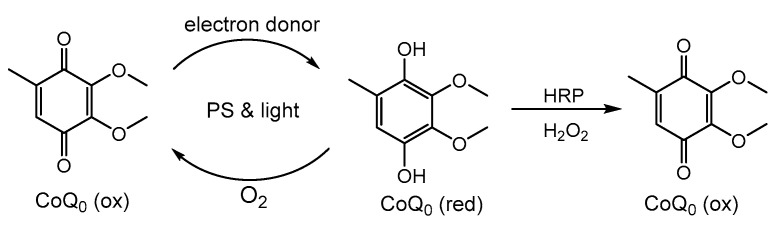
Cycles of CoQ_0_ photoreduction and photo-oxidation; detection of H_2_O_2_ with horseradish peroxidase (HRP); PS = photosensitizer.

If a catechol were oxidized to an o-quinone and subsequently reduced back to the catechol photochemically, we postulated that a stable p-benzoquinone could be photoreduced to the hydroquinone and photo-oxidized back to the quinone form. Indeed, we reported that cycles of photo-oxidation and photoreduction of the p-benzoquinone CoQ_0_ occurred and that H_2_O_2_ is generated as a byproduct (Scheme 1) [15]. CoQ_0_ is a more water-soluble analog of ubiquinone that only lacks a hydrophobic isoprenyl group [4,5]. Other p-benzoquinones reacted in the same manner, and photochemical cycling and H_2_O_2_ generation occurred regardless of the photosensitizer, MB or pheoA [15].

To expand this work, both o- and p-naphthoquinones were selected as photochemical substrates employing the photosensitizers MB and pheoA. We were particularly interested in menadione because all forms of vitamin K contain the same p-naphthoquinone core [16,17]. Menadione is vitamin K_3_, and to function in blood coagulation, vitamin K must be reduced; a photoreduction pathway for this has not been explored [18]. Herein, our objectives were three-fold: (1) to examine the photoreduction of stable o-naphthoquinones in support of our work on dopamine o-quinone photoreduction; (2) to explore photochemical redox cycling of o- and p-naphthoquinones; and (3) to elucidate the influence of O_2_ concentrations on quinone photoreduction rates.

## 2. Results

We reported that p-benzoquinones can be photoreduced by the combination of photosensitizers, electron donors and red light [15]. Because H_2_O_2_ was generated concurrently, we determined that the newly reduced hydroquinone was reacting with ^1^O_2_ to re-oxidize it back to the p-quinone. Evidence supports H_2_O_2_ generation via ^1^O_2_-mediated hydrogen atom abstraction from the hydroquinone to yield a peroxyl radical (•OOH) and a semiquinone. The pK_a_ of •OOH is 4.8; therefore, at neutral pH, it will deprotonate to superoxide anion [19]. The disproportionation of two superoxide anions yields O_2_ and H_2_O_2_ according to Equation (1). Further, two semiquinones (RO•) disproportionate to yield a hydroquinone and regenerate a p-benzoquinone [20].

O_2_^−•^ + O_2_^−•^ + 2H^+^ → H_2_O_2_ + O_2_ (1)

H_2_O_2_ was quantitated using horseradish peroxidase (HRP), an enzyme that uses H_2_O_2_ to oxidize many organic molecules, including catechols and hydroquinones [21,22,23]. Because hydroquinones are oxidized by HRP only when H_2_O_2_ is available, the p-quinone concentration increased when HRP was added after irradiation (Scheme 1). For CoQ_0_, we monitored the loss of absorbance at 405 nm for photoreduction and the subsequent increase in A_405_ when HRP was added. When catalase was added prior to HRP, no increase in A_405_ was detected because it consumes H_2_O_2_ according to Equation (2):

2H_2_O_2_ → 2H_2_O + O_2_ (2)

**Scheme 2:**
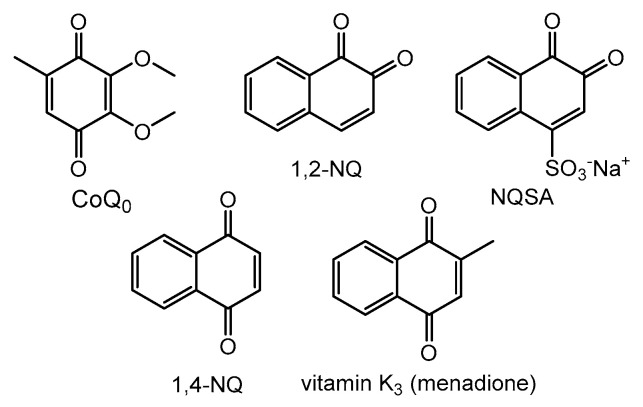
Quinone substrates.

The methodology described above for benzoquinones was applied herein to stable o-naphthoquinones including 1,2-naphthoquinone (1,2-NQ) and 1,2-naphthoquinone sulfonic acid (NQSA) (Scheme 2).

As in our prior work, we employed the combination of methylene blue (MB) as a photosensitizer and EDTA as the tertiary amine electron donor. Importantly, we initiated this work by studying the photoreduction time course in a 1 mL quartz cuvette where it could be irradiated and scanned directly at time intervals to avoid mixing and limit O_2_ exposure to only what was in the solution.

**Figure 1:**
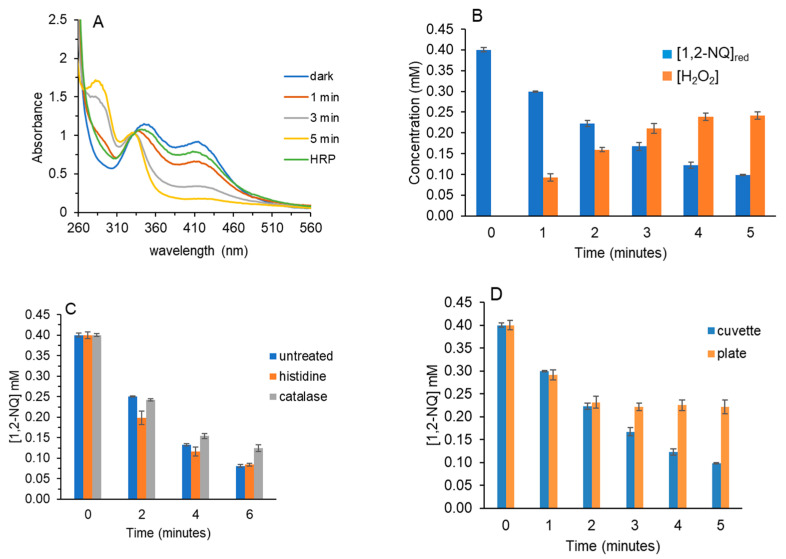
Photoreduction of 1,2-NQ by MB/EDTA. (**A**) Reactions (1 mL) contained 0.4 mM 1,2-NQ, 2 μM MB and 2 mM EDTA in 10 mM PB pH 7.4 (2% DMF final). UV/Vis scans were collected prior to (dark) and after irradiation. These data are the average of at least three independent experiments. HRP (1 μM final) was added after 5 min of light exposure and re-scanned. (**B**) Samples were prepared as in (**A**). The decrease in A_415_ nm was used to calculate [1,2-NQ] at each time. The increase in A_415_ after HRP addition was used to calculate [H_2_O_2_] (post irradiation). These data are the average of at least three independent experiments. (**C**) Samples were prepared as in (**A**). For treatments, 1 mM histidine or 1 μM catalase was included prior to irradiation. These data are the average of two independent experiments performed in triplicate. (**D**) Reactions were as in (**A**). Portions of 100 μL were irradiated in a 96-well plate; A_405_ was monitored. The cuvette data are identical to those shown in (**B**). The plate data are the average of three independent experiments performed in triplicate.

Figure 1A shows the absorbance changes that occurred upon irradiation under a red light with maximum output at 660 nm. 1,2-NQ has two absorbance peaks at 350 nm and 415 nm, both of which decreased as it was photoreduced. This was apparent because the visible yellow color of 1,2-NQ decreased. As irradiation time increased, absorbance at both 350 nm and 415 nm decreased. In the absence of MB or EDTA, no photoreduction was observed.

Supplemental Figure S1 also includes a peak at 665 nm corresponding to MB. It is important to monitor changes in MB because photobleaching can occur when light is excessive. After 5 min of irradiation, HRP was added to determine if H_2_O_2_ was formed and if reduced 1,2-NQ was a co-substrate for HRP. Figure 1A shows that the absorbance peaks of 1,2-NQ increased after HRP was added, which is consistent with our prior work using p-benzoquinones.

An isosbestic point at 335 nm indicates photoreduction to only one other species, the reduced hydroquinone. In support of this, we also mixed 1,2-NQ directly with the chemical reducing agents sodium borohydride (NaBH_4_) or ascorbate. Supplemental Figure S2 shows that treatment of 1,2-NQ with NaBH_4_ resulted in complete loss of the absorbance at 415 nm. Therefore, we can measure the decrease at 415 nm to determine the moles of 1,2-NQ photoreduced per mole of photosensitizer, which is reported as the turnover number in photochemical studies.

Also, the change at 415 nm was used to determine the concentration of H_2_O_2_ that formed during irradiation. Supplemental Figure S1 shows the change in 1,2-NQ at all 1-min intervals vs. only 1, 3 and 5 min in Figure 1A (omitted for clarity). These values were converted to [1,2-NQ] and plotted vs. time in Figure 1B (in blue). Multiple photoreduction experiments were performed to determine [H_2_O_2_] at each time interval. For example, a separate sample was irradiated for 4 min, scanned and then treated with HRP to determine the concentration of H_2_O_2_ after 4 min. H_2_O_2_ concentration values are shown in Figure 1B (orange) at each time.

According to our proposed mechanism, for each hydroquinone that reacts with ^1^O_2_, one H_2_O_2_ molecule is formed. Because the typical concentration of dissolved O_2_ at 20–22 °C is ~0.3 mM, the maximum concentration of H_2_O_2_ that can form is 0.3 mM if no additional O_2_ enters the reaction vessel. The dimensions of the 1 mL cuvette limit O_2_ from mixing with the solution. Figure 1B shows that H_2_O_2_ increased to ~0.25 mM at 4 min and remained constant at 5 min. Once O_2_ was largely depleted, no more H_2_O_2_ could form. At 5 min, ~75% of the 0.4 mM 1,2-NQ was photoreduced, and some photobleaching of MB is observed in Supplemental Figures S1 and S2.

The crucial role of dissolved O_2_ concentration during photoreduction was explored in Figure 1C. Either catalase or histidine was included in the same 1,2-NQ photoreduction reactions with MB and EDTA as described for Figure 1A,B. By regenerating O_2_ from H_2_O_2_ according to Equation (2), catalase favors the formation of more ^1^O_2_, which will subsequently re-oxidize 1,2-NQ hydroquinone. Indeed, catalase treatment slowed the rate of 1,2-NQ photoreduction at 4 and 6 min but not at 2 min (Figure 1C gray vs. blue). At 2 min, dissolved O_2_ is still present and could be photoexcited to ^1^O_2_. This is apparent from the time course of H_2_O_2_ formation from dissolved O_2_ that is shown in Figure 1B.

Histidine is a well-characterized ^1^O_2_ scavenger, and it reacts with ^1^O_2_ to form an oxygenated species, oxo-histidine, thereby removing O_2_ from the solution [24,25,26]. Consequently, Figure 1C shows that the rate of 1,2-NQ photoreduction increased at 2 and 4 min when 1 mM histidine was present. With less O_2_ available, re-oxidation of the 1,2-NQ hydroquinone would be limited. Decreased H_2_O_2_ for histidine-treated samples was confirmed because, when HRP was added to histidine samples after irradiation, the resultant increase in A_415_ was ~half that of untreated samples.

We tested azide as a ^1^O_2_ scavenger but did not observe the same effect as for histidine treatment. Azide reacts avidly with ^1^O_2_, but O_2_ is regenerated, and additional radical intermediates form [27,28]. For these reasons, we did not pursue azide studies further.

The dependence on O_2_ concentration observed in Figure 1C led us to directly compare the rate of 1,2-NQ photoreduction in the 1 mL cuvette vs. in a 96-well plate. Our early work on catechol photo-oxidation was performed exclusively in a plate format with only 100 µL per well to avoid O_2_ depletion [14]. MB, EDTA and 1,2-NQ solutions were premixed and then aliquoted into a cuvette (1 mL) or the plate wells (100 µL each). For the plate readings, a 405 nm filter was used vs. A_415_ in Figure 1A–C. Figure 1D shows that the reaction format had a profound effect on the rate of 1,2-NQ photoreduction. The photoreduction of 1,2-NQ leveled off after only 2 min in the plate, whereas it continued to decrease in the cuvette. Longer times (past 5 min) had no effect on 1,2-NQ absorbance in the plate. We attribute this stark difference to the increased exposure to O_2_ in the plate. In addition to increased ^1^O_2_ formation, the newly reduced 1,2-NQ is also exposed to O_2_ at the well/air interface and may be directly oxidized by O_2_ independent of light-generated ^1^O_2_.

When HRP was added to select wells to determine the H_2_O_2_ concentration, A_405_ values increased up to the starting values for 1,2-NQ (dark values at t = 0 min). Because the HRP assay for H_2_O_2_ is contingent on having sufficient amounts of reduced 1,2-NQ to react with H_2_O_2_ (1:1), it is not possible to accurately measure the H_2_O_2_ concentration if A_405_ increases to the starting value. Some H_2_O_2_ may have remained even though all the 1,2-NQ was re-oxidized (co-substrate limited). The addition of a second co-substrate is complicated by the formation of multiple one-electron-oxidized intermediates in HRP-catalyzed oxidation [20].

Lastly, 1,2-NQ was photoreduced in a glass vial to allow for O_2_ uptake measurements. In this format, ~90% of the dissolved O_2_ was depleted (~0.27 mM), and yet only 25–30% of the 1,2-NQ was reduced. The extent of reduction was based on the same absorbance decrease at 415 nm. However, to measure absorbance, a portion of the solution had to be transferred to a cuvette, which likely resulted in some air oxidation.

**Figure 2:**
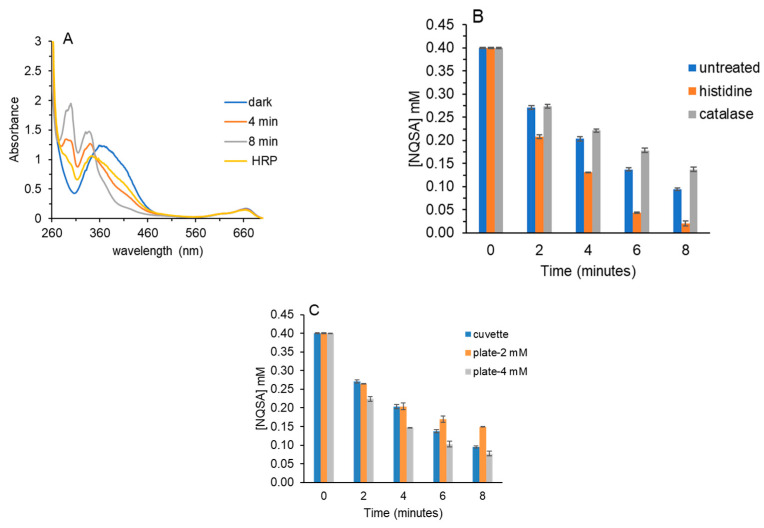
Photoreduction of 1,2-NQSA by MB/EDTA. (**A**) Reactions (1 mL) contained 0.4 mM 1,2-NQSA, 2 μM MB and 2 mM EDTA in 10 mM PB pH 7.4 (2% DMF final). UV/Vis scans were collected prior to (dark) and after irradiation. HRP (1 µM final) was added after 8 min light of exposure and re-scanned. The decrease in A_405_ nm was used to calculate [1,2-NQSA] at each time. The increase in A_405_ after HRP addition was used to calculate [H_2_O_2_] (post irradiation). (**B**) Samples were prepared as in (**A**). Histidine (1 mM) or 1 µM catalase was included prior to irradiation. These data are the average of two independent experiments performed in triplicate. (**C**) Samples were prepared as in (**A**), and 100 µL portions containing either 2 or 4 mM EDTA were irradiated in a 96-well plate. A_405_ was measured prior to and after light exposure. The cuvette data are identical to that shown in (**B**). These data are the average of three independent experiments performed in triplicate.

The photoreduction of a sulfonic acid derivative of 1,2-NQ (NQSA) was studied in the same manner. Figure 2A shows the UV/Vis spectrum of NQSA prior to and after irradiation. Instead of two peaks at 350 and 415 nm like 1,2-NQ (Figure 1A), NQSA has a broader peak ~370 nm that extends into the visible range. As for 1,2-NQ, a loss of yellow coloration was apparent upon photoreduction. Two new peaks in the UV attributed to the NQSA hydroquinone increased as time increased. The addition of HRP after 8 min of total light exposure re-oxidized reduced NQSA, and the UV peaks decreased as the visible absorbance increased. The H_2_O_2_ concentration was 0.26 ± 0.05 mM, which is in excellent agreement with the concentration formed during 1,2-NQ photoreduction (Figure 1B).

As for 1,2-NQ, we added a chemical reducing agent to determine the spectrum of fully reduced NQSA. Supplemental Figure S3 shows that the addition of ascorbate decreased absorbance to the baseline at 400–410 nm and, in that region, overlaid exactly with NQSA that had been photoreduced. Of note, ascorbate itself has a strong absorbance below 300 nm, which is evident in Supplemental Figure S3. When NaBH_4_ was employed to chemically reduce NQSA, the same complete loss at 410 nm was observed.

In Supplemental Figure S3, 4 mM EDTA was used, but only 2 mM EDTA was used in Figure 2A. Thus, for the complete photoreduction of 0.4 mM NQSA in 8 min, more EDTA (electron donor) was required. For comparison, complete photoreduction was observed after 6 min with 2 mM EDTA for 0.4 mM 1,2-NQ.

Figure 2B shows that NQSA photoreduction was also affected by catalase or histidine. As seen in Figure 1C for 1,2-NQ, catalase slowed the observed rate of NQSA photoreduction at 4, 6 and 8 min when more O_2_ would have been depleted, and it was regenerated via the catalase reaction with H_2_O_2_ (Figure 2B blue vs. gray). By increasing O_2_, more ^1^O_2_ is likely to form, thereby oxidizing newly photoreduced NQSA and slowing the observed rate of photoreduction. Histidine increased the rate of NQSA photoreduction at all times. All concentrations of MB, EDTA, quinone and histidine were identical to those in Figure 1C. Yet, the effect of histidine on NQSA photoreduction in Figure 2B is greater than that for 1,2-NQ in Figure 1C.

Figure 2C shows that the rates of NQSA photoreduction in the cuvette vs. plate formats were also different. Two EDTA concentrations (2 vs. 4 mM) were employed to determine if that affected the rate and extent of photoreduction in a 96-well plate as it did in the cuvette format (Figure 2A vs. Supplemental Figure S3). At 2 and 4 min, cuvette vs. plate values (both with 2 mM EDTA) were equivalent, but at 6 and 8 min, the cuvette rate surpassed that of the plate. This is consistent with greater O_2_ exposure in the wells, while we know that, in the cuvette, O_2_ would have been depleted at longer times (Figure 1B). By contrast, the plate well reactions with 4 mM EDTA proceeded to a greater extent.

**Figure 3:**
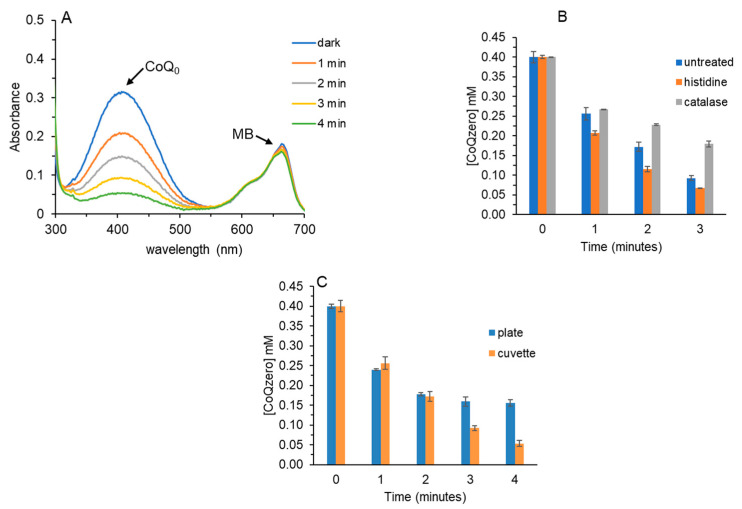
Photoreduction of CoQ_0_ by MB/EDTA. Reactions (1 mL) contained 0.4 mM CoQ_0_, 2 μM MB and 2 mM EDTA in 10 mM PB pH 7.4 (2% DMF final). (**A**) UV/Vis scans were collected prior to (dark) and after irradiation. (**B**) Samples were prepared as in (**A**). Histidine (1 mM) or 1 μM catalase were included prior to irradiation. The decrease in A_405_ was used to calculate [CoQ_0_] at each time. These data are the average of two independent experiments performed in triplicate. (**C**) Samples were prepared as in (**A**), and 100 µL portions were irradiated in a 96-well plate. A_405_ was measured prior to and after light exposure. The cuvette data are identical to that shown in (**B**). These data are the average of three independent experiments performed in triplicate.

Although our prior work focused on CoQ_0_ photoreduction, we did not examine O_2_ dependence to the same extent as described here for the o-naphthoquinones [15]. Therefore, we revisited CoQ_0_ photoreduction using the same reaction conditions (0.4 mM quinone, 2 μM MB and 2 mM EDTA) as for 1,2-NQ. Figure 3A shows the light-dependent decrease in CoQ_0_ absorbance as it was reduced to hydroquinone. After 4 min, 83% of the CoQ_0_ was reduced, and only minimal photobleaching was detected (Figure 3A). From the increase in CoQ_0_ absorbance at 405 nm after HRP addition, we calculated a H_2_O_2_ concentration of 0.26 ± 0.03 mM (3 min light treatment).

As for 1,2-NQ and NQSA, we examined the effects of catalase and histidine on the rates of CoQ_0_ photoreduction (Figure 3B). Again, catalase slowed the observed rate of photoreduction at 2 and 3 min when more O_2_ would have been depleted and catalase regenerated it. Histidine (1 mM) increased the rate of CoQ_0_ photoreduction by scavenging ^1^O_2_, thereby limiting hydroquinone re-oxidation.

A comparison of CoQ_0_ photoreduction in both the cuvette and the plate formats shows that the rates were identical at 1 and 2 min (Figure 3C). However, at 3 and 4 min, further photoreduction in the plate did not occur, while it continued in the cuvette (Figure 3A). This is consistent with the results for 1,2-NQ (Figure 1D) and, to a lesser extent, with the NQSA results (Figure 2C).

**Figure 4:**
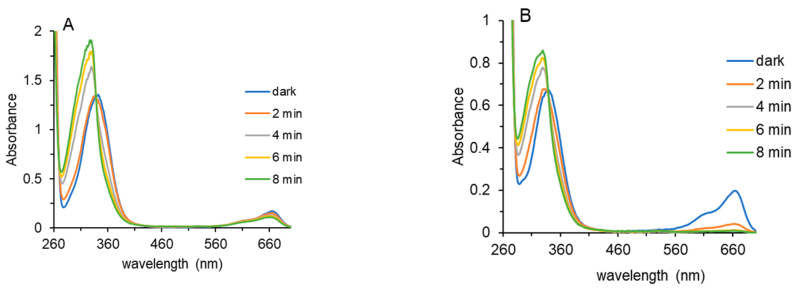
Photoreduction of 1,4-NQ and menadione by MB/EDTA. (**A**) Reactions (1 mL) contained 0.4 mM 1,4-NQ, 2 μM MB and 2 mM EDTA in 10 mM PB pH 7.4 (2% DMF final). (**B**) Reactions (1 mL) contained 0.25 mM menadione, 2.5 μM MB and 2.5 mM EDTA in 10 mM PB pH 7.4. UV/Vis scans were collected prior to (dark) and after each irradiation time.

The photoreduction of the p-naphthoquinones 1,4-NQ and menadione was also examined by UV/Visible spectroscopy prior to red light exposure and at select times. Neither absorbs in the visible range, as seen in the spectrum of 1,4-NQ in Figure 4A. A peak at 338 nm for the oxidized quinone shifted to a larger one at 330 nm for the 1,4-NQ hydroquinone. An isosbestic point is observed, indicative of conversion between those two species. After irradiation for 2 min, there was little change in the spectra (Figure 4A). At longer times, the shift to the reduced form is apparent. When ascorbate was added after 8 min, the spectra overlaid with those of the 8 min sample, confirming that 1,4-NQ was photoreduced completely after 8 min. The addition of HRP yielded a shift back to the oxidized form; however, given the close peaks of oxidized and reduced 1,4-NQ, quantitation was more challenging. We estimated a H_2_O_2_ concentration of ~0.3 mM.

For menadione, similar spectra were observed for the oxidized and reduced forms. Figure 4A shows that irradiation induced a peak shift and increase that correspond to the reduced form. Of note, for menadione, the MB peak at ~665 nm decreased by 80% after only 2 min and remained low at all times. Although some MB photobleaching is observed in Figure 4A, it is not as striking as shown for menadione in Figure 4B. As for all quinones tested, the addition of HRP regenerated oxidized menadione.

Table 1 summarizes the conditions employed to photoreduce the five quinones with MB and EDTA. The turnover number (mol quinone reduced/mol MB), the ratio of EDTA to MB and the time for complete reduction are included. CoQ_0_, a benzoquinone, was the easiest to reduce, requiring only 4 min and a lower ratio of EDTA:MB of 1000. The o-naphthoquinones, 1,2-NQ and NQSA, were more difficult to reduce as evidenced by the longer irradiation times, and for NQSA, a higher ratio of EDTA:MB was required. 1,4-NQ was nearly equivalent to 1,2-NQ. Of the five, menadione was the most difficult to photoreduce, requiring a higher concentration of MB (lower turnover number) and a higher ratio of EDTA to MB. The effect of the menadione methyl group on photoreduction is noteworthy.

Further data analysis for 1,2-NQ, NQSA and CoQ_0_ was performed to obtain rate constants and half-lives for the reaction conditions presented in Figure 1C, Figure 2B and Figure 3B. Clear differences in photoreduction rates are evident with the largest rate constant and consequently the shortest half-life for CoQ_0_, followed by 1,2-NQ and NQSA. For all three quinones, rate constants increased with histidine treatment and decreased with catalase. Given the overlapping absorbance peaks of oxidized and reduced 1,4-NQ and menadione, we did not calculate rate constants (Table 2).

The chlorophyll metabolite pheophorbide A (pheoA) is of great interest as a photosensitizer for quinone photoreduction because it is the species that is formed from dietary chlorophyll [29,30,31]. In our prior work with pheoA (with one carboxylate), EDTA was not an effective electron donor due to its multiple carboxylates at pH 7.4 [14]. For that reason, triethanolamine (TEOA), with its pKa of 7.75 and no charges, is used as the electron donor with pheoA. However, even with TEOA as the electron donor and no charge repulsion, pheoA turnovers (mol quinone reduced/mol pheoA) were consistently lower than MB (~50 vs. up to 300). For optimized pheoA solubility and reactivity, 20% DMF was included in all pheoA-mediated photochemical reactions. We did not test higher DMF concentrations because we did not want to compromise the activity of HRP and catalase, enzymes that we used to elucidate photo-oxidation and photoreduction pathways.

**Figure 5:**
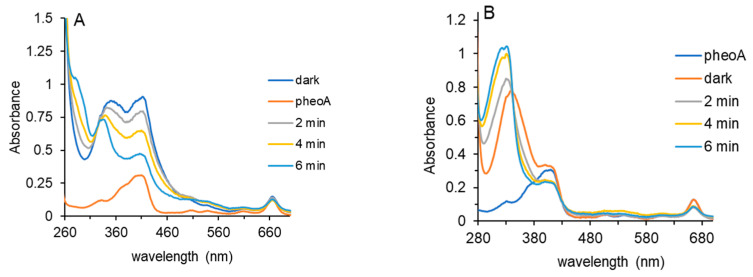
Photoreduction of 1,2-NQ and menadione by pheoA/TEOA. Reactions contained 0.25 mM 1,2-NQ (**A**) or menadione (**B**), 5 μM pheoA, and 10 mM TEOA in 10 mM PB pH 7.4 (20% DMF final). UV/Vis scans were collected prior to (dark) and after each irradiation time.

Figure 5A shows the UV/Vis spectra of pheoA (5 μM), 1,2-NQ (0.25 mM) prior to irradiation and after 2, 4 and 6 min of light treatment. PheoA has a broad absorbance centered ~410 nm that overlaps with 1,2-NQ (Figure 1A). Therefore, only 0.25 mM 1,2-NQ was used in photoreduction reactions with pheoA to ensure that the absorbance values did not exceed the linear range. The photoreduction of 1,2-NQ is evident from the absorbance decreases at 415 and 350 nm, from the isosbestic point and from the increase in absorbance in the UV range. Photobleaching of pheoA, observable at 665 nm, did not occur over this time range. As for MB-mediated photoreduction assays, the addition of HRP increased the absorbance of the oxidized o-naphthoquinone.

The menadione photoreduction spectra are shown in Figure 5B. As in Figure 5A, the spectrum of pheoA is included. Because the absorbance maxima of oxidized and reduced menadione are at lower wavelengths than pheoA, the photoreduction of menadione is easier to observe than for 1,2-NQ (Figure 5A). With only 2 min of light treatment, the photobleaching of pheoA is observed with decreases in absorbance at ~410 nm and at 665 nm. No further decreases in pheoA absorbance occurred at 4 or 6 min. The photoreduction of menadione was complete after 6 min. Identical concentrations of quinone (1,2-NQ or menadione), pheoA and TEOA were used for the experiments shown in Figure 5A,B. Menadione was photoreduced to a greater extent than 1,2-NQ.

CoQ_0_, NQSA and 1,4-NQ were also photoreduced using the same combination of pheoA/TEOA and 20% DMF, and the results are summarized in Table 3. UV/Vis scans for CoQ_0_, NQSA and 1,4-NQ are shown in Supplemental Figures S4–S6. The lower turnover number for pheoA relative to MB is apparent and was consistently ~50 (Table 3). For all quinones tested, the reaction conditions that achieved those 50 turnovers were equivalent. This differs from the MB/EDTA results summarized in Table 1, where it is clear that menadione was more difficult to photoreduce than the other quinones.

While UV/Vis scans prior to and after irradiation confirmed that photoreduction occurred, quantitation is challenging especially for the 1,4-naphthoquinones that only absorb in the UV range. Further, chlorophyll metabolites like pheoA absorb at the same wavelengths as many of the quinones we studied. Therefore, we sought an electron acceptor capable of reacting with photoreduced quinones that would yield a color change outside the wavelength range of 300–450 nm.

Tetrazolium salts act as electron acceptors in many biochemical assays, particularly when they are reduced by dehydrogenase enzymes as NADH is oxidized [32,33,34]. Following photoreduction according to the MB/EDTA conditions presented in Table 1, portions of cuvette, microfuge tube and plate well reactions were combined with MTT, a well-characterized tetrazolium salt. Upon mixing, an immediate color change was observed that was consistent with electron transfer from the hydroquinones to MTT. The reduced MTT product, a formazan, was quantitated based on its characteristic absorbance ~600 nm. To calculate the concentration of formazan, we prepared an NADH standard curve. NADH reacts 1:1 with MTT but requires a catalytic amount of phenazine methosulfate (PMS). Although MTT formazan has limited solubility, no precipitates were observed during the time course of these assays (up to 15 min total).

**Figure 6:**
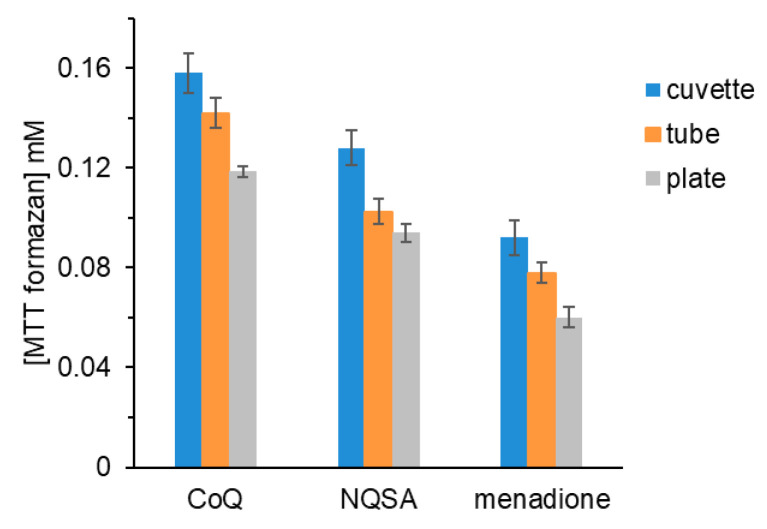
Detection of photoreduced quinones with MTT. Photoreduction reactions were prepared with MB/EDTA as summarized in Table 1. Aliquots of reduced quinones (40 µL) were combined with 60 µL 2 mM MTT in a 96-well plate. Absorbance at 562 and 630 nm (MTT formazan) was measured. These data are the average of three samples performed in duplicate for each of the following: a cuvette, tube and plate.

All of the quinones, when photoreduced, yielded a color change when combined with MTT; no PMS was required. Figure 6 shows the results of these experiments for CoQ_0_, NQSA and menadione. Samples of each quinone in the three experimental configurations were photoreduced concurrently. Based on our experimental design, the highest possible formazan concentration was 0.16 mM. For CoQ_0_, that was achieved only in the cuvette with lower formazan yields for CoQ_0_ photoreduced in a microfuge tube or plate well. This trend of highest MTT formazan concentrations in the cuvette, followed by the tube and plate, is evident for NQSA and menadione as well (Figure 6). When hydroquinone, the stable reduced form of 1,4-benzoquinone, was combined with MTT, no reaction occurred. Likewise, samples that contained MB, EDTA and quinones but that were not irradiated did not react with MTT.

The formazan concentrations for NQSA and menadione are lower than those of CoQ_0_. Although the extent of reduction in the cuvette should have approached 100% for NQSA and menadione, mixing with MTT introduced O_2_ that resulted in re-oxidation of a portion of the hydroquinones so that MTT was not reduced to the same extent. Mixing with MTT was identical for CoQ_0_, NQSA and menadione; thus, the differences in MTT reduction represent different sensitivities to O_2_.

## 3. Discussion

The results herein confirm that both o- and p-naphthoquinones are readily photoreduced by the combinations of MB and EDTA as a photosensitizer and electron donor and by pheoA and TEOA. This is consistent with our prior work on several p-benzoquinones including CoQ_0_ [15]. As for p-benzoquinones, the combination of MB and EDTA was superior to pheoA and TEOA with respect to the number of turnovers (~200 for MB and ~ 50 for pheoA), which are summarized in Table 1 and Table 3. Also, for optimal photoreduction, 20% DMF was included for photoreduction with pheoA and TEOA.

**Scheme 3:**
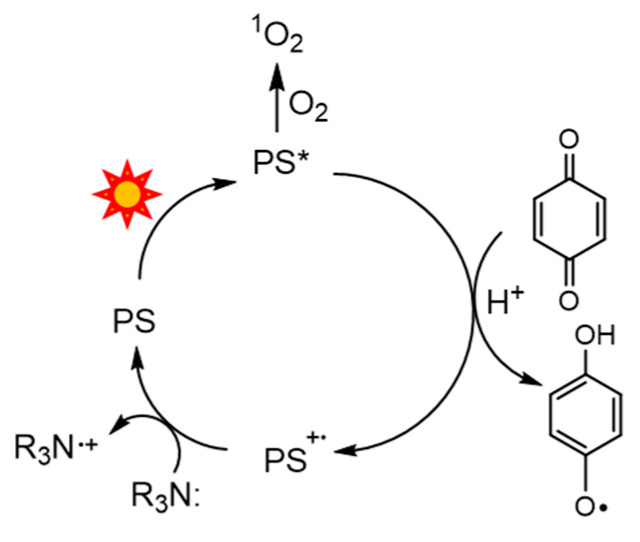
Photochemical oxidation and reduction pathways.

Our data support concurrent naphthoquinone photoreduction and ^1^O_2_-mediated re-oxidation. Once a photosensitizer (PS) is excited by red light to PS*, the triplet state, it may react with O_2_ to generate ^1^O_2_ or transfer an electron to a quinone substrate (Scheme 3). Electron transfer to the quinone yields the radical cation (PS^+•^), which is reduced by a tertiary amine electron donor (R_3_N).

The amount of ^1^O_2_ that forms is dependent on the O_2_ concentration; once O_2_ is depleted, especially in our cuvette experiments, no more ^1^O_2_ can form, and electron transfer to the quinones is favored. ^1^O_2_ is short-lived and may react with the newly generated hydroquinones or with the tertiary amine electron donor according to Equation (3):

^1^O_2_ + R_3_N: → O_2_^−•^ + R_3_N^+•^ (3)

Indeed, we reported that ^1^O_2_ reacts with the tertiary amine electron donors EDTA and TEOA (no quinone present) [15]. When coupled to Equation (1), H_2_O_2_ is produced [35,36].

Even in intact chloroplasts, chlorophyll initiates ^1^O_2_ formation when light intensity is excessive [37]. Thus, chlorophyll functions in both photo-oxidation and photoreduction pathways in living plants.

We detected H_2_O_2_ during the photoreduction of all the naphthoquinones tested, which is consistent with this mechanism of photoreduction followed by the re-oxidation of the resulting hydroquinones when they react with ^1^O_2_ (Scheme 1). The detection of H_2_O_2_ was achieved using HRP, an enzyme that oxidizes a wide range of organic co-substrates only when H_2_O_2_ is present [21,22]. This work confirms that reduced naphthoquinones are HRP co-substrates.

Our data indicate that the bulk of the H_2_O_2_ produced during photoreduction resulted from the reaction of ^1^O_2_ with hydroquinones, not with R_3_N electron donors. When photoreduction is first initiated, no hydroquinones are present, and ^1^O_2_ may form and react with electron donors to yield some H_2_O_2_. However, the rate constant for ^1^O_2_ and tertiary amines is several orders of magnitude lower than that of hydroquinones and ^1^O_2_ [35,38]. Further, lower EDTA and TEOA concentrations were used herein; for pheoA reactions, the highest TEOA concentration was 16 mM (Figure 5 and Table 3) vs. 50 mM TEOA in prior work.

According to Scheme 3, electron flow for quinone reduction can be enhanced by increasing the electron donor concentration (R_3_N) [39]. This was observed in Figure 2C and Table 1, where higher EDTA concentrations increased the extent of photoreduction. For NQSA in Figure 2, either 2 or 4 mM EDTA was used; samples with 4 mM EDTA yielded the same or less H_2_O_2_ as those that contained 2 mM EDTA. If ^1^O_2_ were reacting with EDTA, we would have expected more H_2_O_2_.

For the experiments in Figure 1C, Figure 2B and Figure 3B, 1 mM histidine was used to scavenge ^1^O_2_ and trap dissolved O_2_ in the process. As a result, H_2_O_2_ concentrations decreased ~50% in those samples with histidine. In these experiments, histidine, EDTA and hydroquinones may all be competing for reaction with ^1^O_2_. The reported rate constant for histidine and ^1^O_2_ is ~10^7^ M^−1^ s^−1^, which is two orders of magnitude greater than that of ^1^O_2_ and EDTA [25,26,35]. Thus, it is likely that histidine and hydroquinone are competing because their published rate constants are comparable (10^7^ vs. 10^8^ M^−1^ s^−1^, respectively) [38].

A clear trend is observed in Table 1 and Table 2 regarding the ease of photoreduction by MB, EDTA and red light. The p-benzoquinone CoQ_0_ is easiest to reduce followed by the o-naphthoquinones and then the p-naphthoquinones. This trend matches the reported differences in reduction potentials [40,41,42]. Based on these reports, o-quinones are more readily reduced than p-quinones, and with the addition of an aromatic ring in the naphthoquinones, reduction becomes more difficult.

Further, the photochemical outcome is dependent on the ease of electron transfer between reaction components and the solubility of the quinone substrates. In the case of NQSA, its sulfonic acid substituent will be negatively charged at pH 7.4; therefore, electron transfer to it may be hindered due to charge repulsion. This likely explains why 1,2-NQ, which lacks a sulfonate substituent, was easier to photoreduce than NQSA (Scheme 2 and Table 1). We believe the more efficient electron transfer from EDTA to MB is responsible for the higher turnover numbers relative to pheoA and TEOA (Table 1 vs. Table 3). MB has a positive charge, and EDTA has multiple negative charges at pH 7.4. Also, electron transfer from hydrophobic pheoA may be easier when the quinone is also hydrophobic. All naphthoquinones tested with pheoA and TEOA yielded identical turnover numbers (Table 3).

The critical role of O_2_ concentrations was evident in Figure 1, Figure 2 and Figure 3 where 1,2-NQ, NQSA and CoQ_0_ photoreduction assays were performed in different reaction vessels. In a deep, narrow cuvette, once O_2_ is consumed and oxidized to H_2_O_2_, little additional O_2_ can enter; consequently, the rate of photoreduction is the fastest. In the well of the plate, more O_2_ is available at the solution/air interface. This results in a slowed rate of photoreduction; indeed, for 1,2-NQ, no further reduction occurred in the plate after 2 min of light (Figure 1D).

We employed a novel assay to detect electron transfer from photoreduced quinones to MTT (Figure 6). Tetrazolium salts are used extensively in biochemical assays, and MTT, in particular, is used in cell-based assays to measure toxicity. Tetrazolium salts are routinely used to quantify dehydrogenase reactions; we used nitroblue tetrazolium as part of a redox stain for lactate dehydrogenase activity on native gels [32,33,34]. The results in Figure 6 are notable because the same trend of greater photoreduction in the cuvette vs. the microfuge tube or the plate well is observed. Although some re-oxidation is unavoidable when MTT and reduced quinone solutions are mixed, this is an innovative method for studying photoreduced benzoquinones and naphthoquinones that are susceptible to re-oxidation.

Our results employing pheoA are relevant because it is the likely metabolite formed in vivo after eating green plants and therefore has health implications (Figure 5 and Table 3) [29,30]. Multiple studies in vivo support a role for the combination of red light and chlorophyll in biochemical pathways like the mitochondrial ETC [3,6,7]. While tertiary amines are not physiological electron donors, understanding the photochemical reactions of chlorophyll metabolites is valuable. Even if electron donors are limited in vivo, a single photoreduction event by a chlorophyll metabolite may be sufficient to increase antioxidant capacity or facilitate the reduction of ubiquinone.

Further, menadione is vitamin K_3_, and all other forms of vitamin K contain the same methyl-substituted naphthoquinone core. Green plants like spinach and kale contain high concentrations of both chlorophyll and vitamin K [17]. The reduced form of vitamin K plays a critical role in blood coagulation. While enzymes that reduce it have been identified, a pathway involving its photoreduction is intriguing. Beyond blood coagulation, vitamin K derivatives are involved in calcium homeostasis and function as cellular antioxidants [16,18].

Lastly, this work lends further support for a photochemical method to generate H_2_O_2_. Herein, we generated H_2_O_2_ using MB, EDTA, mild red light, commercially available naphthoquinones and ambient O_2_ in a neutral aqueous solution. All quinones tested to date by our lab exhibit the same behavior of photoreduction followed by the photo-oxidation of the resulting hydroquinone to produce H_2_O_2_. The current H_2_O_2_ industrial process relies on a Pd catalyst, H_2_ and O_2_ gases, anthraquinone as a redox cycler and a mixture of organic solvents [43,44]. Both EDTA and MB are very water-soluble and nontoxic, and our system requires only 2% DMF for naphthoquinone solubility. Thus, our research on the photochemical reactions of chlorophyll metabolites and methylene blue has both health significance and value for the essential industrial process of H_2_O_2_ synthesis.

## 4. Materials and Methods

All chemicals were from Fisher Scientific or Sigma (St. Louis, MO, USA) and were of the highest purity available. PheoA and all quinone stock solutions were prepared in DMF and used immediately or stored at −20 °C for up to two weeks. All other solutions were prepared in water or 10 mM PB pH 7.4. EDTA and TEOA stock solutions were adjusted to 7.4 or 8.0 with NaOH or HCl. Phosphate buffers were equilibrated to room temperature (20–22 °C) to ensure no variation in dissolved O_2_.

Red light specifications. A 36-watt red light composed of 18 2-watt LEDs was used for all photochemical experiments. The maximum wavelength of the emitted light was 660 nm. The intensity was quantitated in lux, and the intensity (as a function of the distance from the light source to the samples) was measured to ensure consistent exposure.

Experimental configurations for the photoreduction of naphthoquinones and CoQ_0_. Experiments were performed in (1) a quartz semimicro cuvette (1 mL), (2) a microfuge tube (100 µL), (3) a 96-well plate (100 µL) or (4) a 15 mL glass vial (5 mL).

Oxygen uptake assays. Reactions (5 mL) contained 0.4 or 1 mM quinone, MB and EDTA in 10 mM PB pH 7.4 in a 15 mL glass vial. Dissolved oxygen was measured prior to light exposure and at the indicated time intervals using a Mettler-Toledo 605-ISM (Columbus, OH, USA) polarographic dissolved oxygen probe. Reactions were performed under ambient oxygen, and the surface of the solution was exposed to air. The maximum amount of DMF was 5% to minimize damage to the electrode components. Absorbance (100 µL or 1 mL portions) was measured prior to and immediately after light exposure. For CoQ_0_, absorbance at 405 nm was used to calculate changes in concentration. Based on scans of known concentrations of CoQ_0_, we determined a molar absorptivity of 740 M^−1^ cm^−1^. For experiments with 1,2-NQ, we determined a molar absorptivity of 2400 M^−1^ cm^−1^ at 415 nm.

Quinone photoreduction assays. Quinones were combined with MB and EDTA in one of the experimental configurations: a plate, tube, cuvette or vial. Typical concentrations of quinone, MB and EDTA were 0.4 mM, 2 μM and 2 or 4 mM, respectively, in phosphate buffer pH 7.4. For pheoA/TEOA, 20% DMF was required for optimal quinone photoreduction. PheoA (8 μM) and TEOA (16 mM) were typical concentrations employed to photoreduce 0.4 mM quinone. For the photoreduction of 0.25 mM quinone, 5 μM pheoA and 10 mM TEOA in PB plus 20% DMF were employed. This maintains the same ratio of quinone to the photosensitizer used to quantitate the turnover number. Table 4 summarizes the wavelengths that were monitored for the photoreduction of each quinone substrate.

Red light irradiation times were optimized and were no greater than 8 min to limit the photobleaching of MB or pheoA. Absorbance (full scans in the cuvette or select wavelengths in the 96-well plate) was measured prior to light exposure and at time intervals. Dark samples containing all reactants served as controls. DMF concentrations typically ranged from 15 to 20% for pheoA samples, and the maximum % DMF with MB/EDTA was 5%. Catalase (30 µL 2 mg/mL, 1 μM final) or histidine (1 mM) was added prior to red-light irradiation. Quinone reducing agents including sodium ascorbate or NaBH_4_ was added after dark scans or after the quinones had been photoreduced to test for complete photoreduction (~5 equivalents).

Detection of H_2_O_2_ in photoreduction samples. HRP (10 µL, 2 mg/mL) was added directly into the cuvette solution (1 mL) after irradiation and mixed gently to avoid introducing O_2_. For the 96-well plate reactions, 2 µL HRP solution was added to 100 µL reaction volumes. Absorbance increases indicative of hydroquinone re-oxidation were monitored for 2–5 min until no further increase in absorbance occurred.

Detection of reduced quinones with MTT. Photoreduction reactions were prepared as described above in these configurations: a cuvette, tube or 96-well plate. Aliquots of MTT solution (60 µL, 2 mM) were first added to a 96-well plate. Then, 40 µL of the reduced quinone reactions was pipetted directly into the MTT solution with only minimal mixing (up and down gently twice). Absorbance readings at 562 and 630 nm were collected at 3 min intervals until the MTT formazan absorbance was constant (up to 15 min total). The reaction of NADH with MTT was used to quantitate the reduced MTT formazan. For NADH detection, the 2 mM MTT solution also contained 25 μM PMS. NADH standards ranged from 0 to 200 μM.

Data analysis. For each treatment/condition, at least three independent experiments were performed in duplicate or triplicate. Mean values were calculated for each independent experiment (mean ± error). For figures showing error bars, mean values were averaged and errors were calculated. Details for each are stated in the figure legends. The rate constants in Table 2 were determined by plotting the natural log of the concentrations shown in Figure 1C, Figure 2B and Figure 3B vs. time. Because all the errors in those figures were less than 5%, we did not include errors in our rate constant or half-life calculations.

## 5. Conclusions

The photoreduction of o- and p-naphthoquinones and their subsequent re-oxidation by ^1^O_2_ was studied with the photosensitizers methylene blue and pheophorbide A. The involvement of ^1^O_2_ was confirmed using histidine, a well-characterized ^1^O_2_ scavenger that limits the re-oxidation step, thereby increasing the rates of photoreduction. Catalase treatment slowed photoreduction by regenerating O_2_ from H_2_O_2_, a byproduct of hydroquinone oxidation by ^1^O_2_. This redox cycling mechanism is consistent with our prior work on several benzoquinones including CoQ_0_, a water-soluble analog of ubiquinone, a key redox-active component of the ETC. Herein, we showed the photoreduction of the p-naphthoquinone vitamin K_3_ by pheoA, the major dietary chlorophyll metabolite, suggesting a photochemical pathway for vitamin K reduction that is vital for its role in blood coagulation. This work on dietary chlorophyll photochemistry is intriguing because many natural products that we consume contain quinone functionalities.

## Figures and Tables

**Table 1:** Summary of photoreduction results with MB and EDTA.

Quinone (Q)	Turnovers (mol Q Reduced/mol MB)	EDTA:MB	Irradiation Time (min)
CoQ_0_	162 ± 8	1000	4
1,2-NQ	178 ± 6	1000	6
NQSA	183 ± 10	2000	8
1,4-NQ	186 ± 12	1000	8
Menadione ^1^	94 ± 8	2000	8

^1^ Reactions contained 0.25 mM menadione, 2.5 µM MB and 5 mM EDTA. All other reactions contained 0.4 mM Q, 2 µM MB and either 2 or 4 mM EDTA.

**Table 2:** Photoreduction rate constants and half-lives for quinones treated with histidine or catalase.

Quinone	Treatment	Rate Constant (min^−1^)	Half-Life (min)
1,2-NQ	None	0.28 ^1^	2.5
	Histidine	0.31	2.2
	Catalase	0.22	3.5
NQSA	None	0.18	3.9
	Histidine	0.37	1.9
	Catalase	0.13	5.4
CoQ_0_	None	0.48	1.4
	Histidine	0.59	1.2
	Catalase	0.26	2.7

^1^ Rate constants and half-lives were calculated from the data in Figure 1C, Figure 2B and Figure 3B.

**Table 3:** Summary of photoreduction results with pheoA and TEOA (20% DMF).

Quinone (Q)	Turnovers (mol Q Reduced/mol pheoA)	Irradiation Time (min)
^1^ CoQ_0_	50 ± 3	4
1,2-NQ	43 ± 5	6
NQSA	47 ± 6	6
1,4-NQ	47 ± 3	6
Menadione	48 ± 3	6

^1^ Reactions contained 0.4 mM CoQ_0_, 8 μM pheoA and 16 mM TEOA. All other reactions contained 0.25 mM Q, 5 μM pheoA and 10 mM TEOA.

**Table 4:** Spectroscopic characteristics of the quinone substrates.

Quinone (Q)	Absorbance Monitored (nm)	Isosbestic Point (nm)
CoQ_0_	↓ 405–410	None
1,2-NQ	↓ 415	335
NQSA	↓ 405	348
1,4-NQ	↑ 330	338
Menadione	↑ 331	339

## Data Availability

Data will be made available on request.

## Supplementary Materials

The following supporting information can be downloaded at https://www.mdpi.com/article/10.3390/molecules30061351/s1, Figure S1: 1,2-NQ photoreduction and re-oxidation with HRP; 1 min time intervals; Figure S2: 1,2-NQ photoreduction and full chemical reduction with sodium borohydride; Figure S3: NQSA photoreduction and full chemical reduction with sodium ascorbate; Figure S4: CoQ_0_ photoreduction with pheoA/TEOA in 20% DMF; Figure S5: NQSA photoreduction with pheoA/TEOA in 20% DMF; Figure S6: 1,4-NQ photoreduction with pheoA/TEOA in 20% DMF.

## Abbreviations

The following abbreviations are used in this manuscript: CoQ_0_, 2,3-dimethoxy-5-methyl-p-benzoquinone; EDTA, ethylenediaminetetraacetic acid; HRP, horseradish peroxidase; MB, methylene blue; MTT, 3-(4,5-Dimethylthiazol-2-yl)-2,5-diphenyltetrazolium bromide; NaBH_4_, sodium borohydride; 1,2-NQ, 1,2-naphthoquinone; NQSA, 1,2-naphthoquinone sulfonic acid; 1,4-NQ, 1,4-naphthoquinone; PB, phosphate buffer; pheoA, pheophorbide A; PMS, phenazine methosulfate; TEOA, triethanolamine.
