# Supplementary material for: Photochemical Redox Cycling of Naphthoquinones Mediated by Methylene Blue and Pheophorbide A
Source: Molecules. 2025 Mar 18;30(6):1351. doi: 10.3390/molecules30061351 (PMC11944901; doi:10.3390/molecules30061351)
Supplement: Supplementary file 1 [file molecules-30-01351-s001.zip › molecules-3493655-supplementary.pdf]

SUPPLEMENTAL FIGURES

Figure S1. 1,2-NQ photoreduction and reoxidation with HRP; 1 minute time intervals

Figure S2. 1,2-NQ photoreduction and full chemical reduction with sodium borohydride

Figure S3: NQSA photoreduction and full chemical reduction with sodium ascorbate

Figure S4. CoQ_0 photoreduction with pheoA/TEOA in 20% DMF

Figure S5: NQSA photoreduction with pheoA/TEOA in 20% DMF

Figure S6: 1,4-NQ photoreduction with pheoA/TEOA in 20% DMF

Figure S1**.** 1,2-NQ photoreduction and reoxidation with HRP; 1 minute time intervals

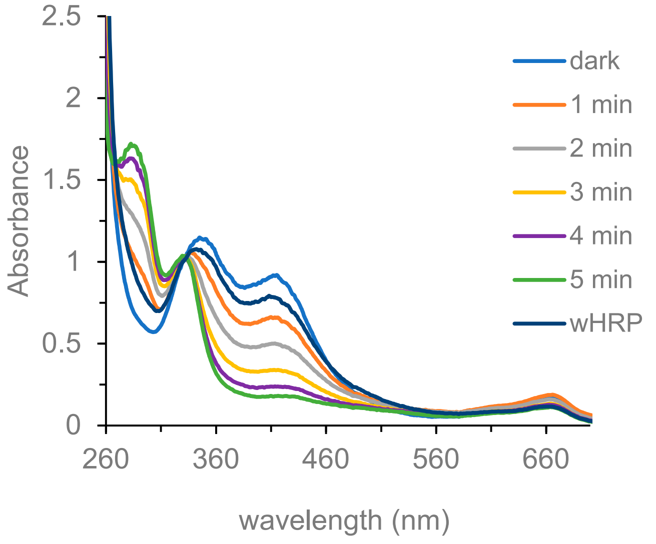

Reactions (1 ml) contained 0.4 mM 1,2-NQ, 2 µM MB and 2 mM EDTA in 10 mM PB pH 7.4 (2% DMF final). UV/Vis scans were collected prior to (dark) and after irradiation. HRP (1 µM final) was added after 5 min light exposure and re-scanned.

Figure S2. 1,2-NQ photoreduction and full chemical reduction with sodium borohydride ($NaBH_4$)

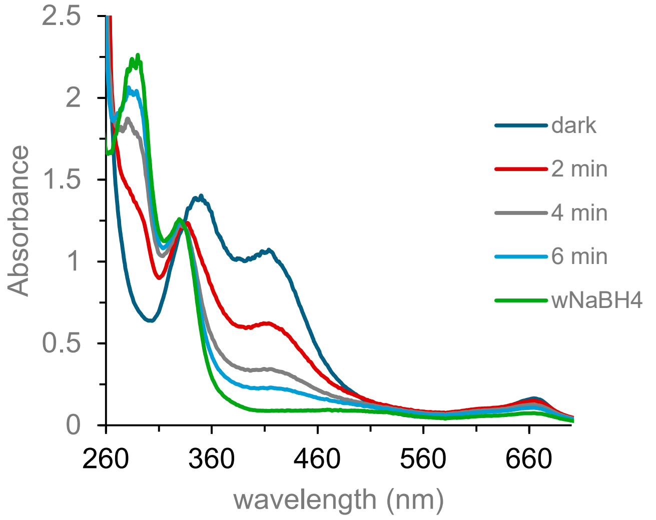

Reactions (1 ml) contained 0.5 mM 1,2-NQ, 2.5 μM MB and 2.5 mM EDTA in 10 mM PB pH 7.4 (2% DMF final). UV/Vis scans were collected prior to (dark) and after irradiation. After 6 min, $NaBH_4$ (~5 equivalents in methanol) was added.

Figure S3: NQSA photoreduction and full chemical reduction with sodium ascorbate

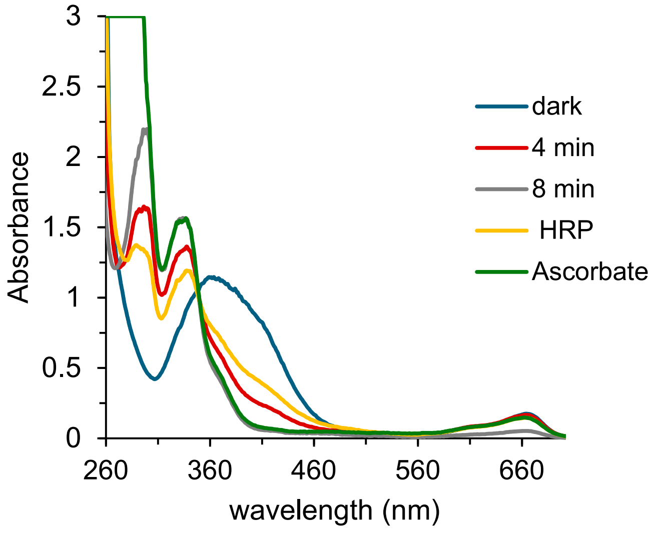

Reactions (1 ml) contained 0.4 mM 1,2-NQ, 2 μM MB and 2 mM EDTA in 10 mM PB pH 7.4 (2% DMF final). UV/Vis scans were collected prior to (dark) and after irradiation. After photoreduction and HRP treatment, sodium ascorbate (2 mM final) was added.

Figure S4. CoQ_0 photoreduction with pheoA/TEOA in 20% DMF

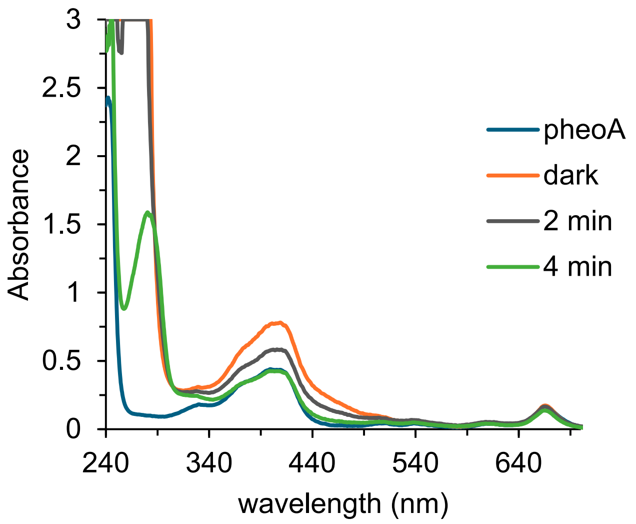

Reactions contained 0.4 mM CoQ_0, 8 µM pheoA and 16 mM TEOA in 10 mM PB pH 7.4 (20% DMF final). UV/Vis scans were collected prior to (dark) and after each irradiation time.

Figure S5: NQSA photoreduction with pheoA/TEOA in 20% DMF

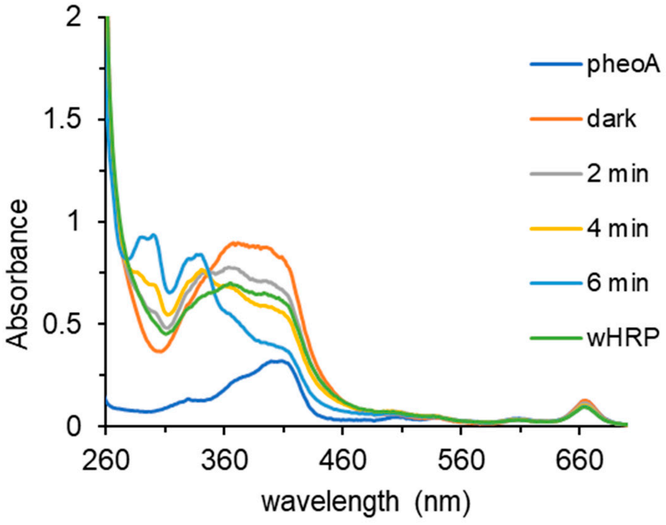

Reactions contained 0.25 mM NQSA, 5 µM pheoA and 10 mM TEOA in 10 mM PB pH 7.4 (20% DMF final). UV/Vis scans were collected prior to (dark) and after each irradiation time. HRP (1 µM final) was added after 6 min light exposure and re-scanned.

Figure S6: 1,4-NQ photoreduction with pheoA/TEOA in 20% DMF

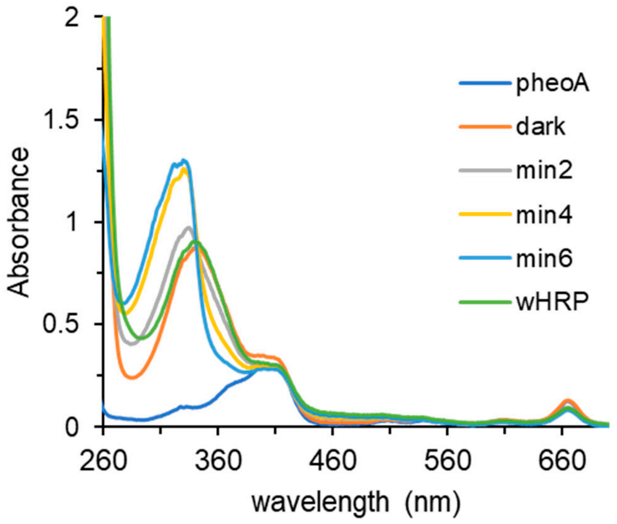

Reactions contained 0.25 mM 1,4-NQ, 5 µM pheoA and 10 mM TEOA in 10 mM PB pH 7.4 (20% DMF final). UV/Vis scans were collected prior to (dark) and after each irradiation time. HRP (1 µM final) was added after 6 min light exposure and re-scanned.
